# Tim50a, a nuclear isoform of the mitochondrial Tim50, interacts with proteins involved in snRNP biogenesis

**DOI:** 10.1186/1471-2121-6-29

**Published:** 2005-07-11

**Authors:** Hongzhi Xu, Z Brad Somers, Melvin L Robinson, Michael D Hebert

**Affiliations:** 1 Department of Biochemistry, The University of Mississippi Medical Center Jackson, MS 39216-4505, USA

## Abstract

**Background:**

The Cajal body (CB) is a nuclear suborganelle involved in the biogenesis of small nuclear ribonucleoproteins (snRNPs), which are vital for pre-mRNA splicing. Newly imported Sm-class snRNPs traffic through CBs, where the snRNA component of the snRNP is modified, and then target to other nuclear domains such as speckles and perichromatin fibrils. It is not known how nascent snRNPs localize to the CB and are released from this structure after modification. The marker protein for CBs, coilin, may play a role in snRNP biogenesis given that it can interact with snRNPs and SMN, the protein mutated in Spinal Muscular Atrophy. Loss of coilin function in mice leads to significant viability and fertility problems and altered CB formation.

**Results:**

In this report, we identify a minor isoform of the mitochondrial Tim50, Tim50a, as a coilin interacting protein. The Tim50a transcript can be detected in some cancer cell lines and normal brain tissue. The Tim50a protein differs only from Tim50 in that it contains an additional 103 aa N-terminal to the translation start of Tim50. Importantly, a putative nuclear localization signal is found within these 103 residues. In contrast to Tim50, which localizes to the cytoplasm and mitochondria, Tim50a is strictly nuclear and is enriched in speckles with snRNPs. In addition to coilin, Tim50a interacts with snRNPs and SMN. Competition binding experiments demonstrate that coilin competes with Sm proteins of snRNPs and SMN for binding sites on Tim50a.

**Conclusion:**

Tim50a may play a role in snRNP biogenesis given its cellular localization and protein interaction characteristics. We hypothesize that Tim50a takes part in the release of snRNPs and SMN from the CB.

## Background

The biogenesis of most spliceosomal small nuclear ribonucleoproteins (snRNPs) is complicated and requires both cytoplasmic and nuclear maturation steps [1-3]. For example, the spliceosomal small nuclear RNAs (snRNAs) of Ul, U2, U4 and U5 snRNPs are synthesized by RNA polymerase II and may traffic through specific subnuclear domains before being exported to the cytoplasm [1,3]. In the cytoplasm, a septet of Sm proteins (B/B', Dl, D2, D3, E, F, G) binds the Sm motif of the snRNA under the control of the Survival of Motor Neurons (SMN) protein complex. Mutations in the SMN protein cause the neurodegenerative disorder Spinal Muscular Atrophy [4,5]. After the Sm core has been assembled onto the snRNA, the snRNA is subject to further processing, followed by import back into the nucleus; again with the help of the SMN complex [1,5-8].

Upon nuclear re-entry, newly assembled Ul, U2, U4 and U5 snRNPs first localize to a subnuclear domain known as the Cajal body [9]. In the Cajal body (CB), the snRNA component of the snRNP is subjected to pseudouridine-base and 2'-*0*-methyl sugar-modifications that are guided by small CB-specific RNAs (scaRNAs) [10-12]. These modifications are crucial for proper pre-mRNA splicing in vivo [13]. After their modification in the CB, snRNPs localize to speckles, where they are stored, or perichromatin fibrils, where splicing occurs concurrently with transcription [14]. Unlike Ul, U2, U4 and U5 snRNAs, maturation of the RNA polymerase III-transcribed U6 snRNA does not include a cytoplasmic phase and may take place in the nucleolus and the CB [3]. The U7 snRNP, which is required for histone pre-mRNA 3'-end processing [15-17], is assembled in manner similar to that observed for Ul, U2, U4 and U5 snRNPs, including a cytoplasmic phase [18]. However, U7 snRNA has a noncanonical Sm binding site and thus recruits a different type of Sm core compared to that which binds Ul, U2, U4 and U5 snRNA [19,20]. Like the spliceosomal snRNPs, the U7 snRNP is enriched within CBs [19,21]. CBs have been shown to move in an ATP dependent manner [22,23] as well as associate with various gene loci, including snRNA and histone gene clusters [24]. Consequently, CBs may provide a platform upon which a feedback regulatory mechanism for snRNP and histone biogenesis takes place [25].

The mechanisms by which snRNPs are targeted to and released from the CB are unknown. One possibility is that a factor within the CB interacts with nascent snRNPs and facilitates their modification. Another factor may displace snRNPs from the CB, allowing for their subsequent localization in speckles and perichromatin fibrils. The CB marker protein coilin may play a role in the targeting of snRNPs to CBs. Removal of coilin in *Xenopus *by immunodepletion decreases snRNP levels in the amphibian equivalent of the CB [26]. Characterization of coilin knockout mice has revealed that they are viable on an outbred mouse strain, but have significant viability and fertility defects on inbred strains [27] (Greg Matera, personal communication). Cell lines derived from coilin knockout mice lack canonical CBs in which snRNPs are enriched [27]. However, add-back experiments demonstrate that typical CBs, containing snRNPs, can be reformed upon the addition of coilin [27]. Furthermore, coilin can interact directly with several Sm proteins of snRNPs [28] (our unpublished observations). Taken together, these data indicate that, while not an essential protein, coilin is important for proper CB formation. Functional CBs may allow for the efficient coordination of the nuclear steps of snRNP biogenesis.

Another protein that requires coilin for its localization to CBs is SMN [27-30]. Coilin directly interacts with SMN via several arginine/glycine (RG) dipeptide repeats in coilin [28]. The arginines within this RG box motif are symmetrically dimethylated [29,30], resulting in an increased affinity for SMN [31,32]. In most cell lines and tissues, SMN localizes to the cytoplasm and CBs [33]. However, SMN in some cell lines and fetal tissue localizes to discrete nuclear structures termed "gems" (for Gemini of Cajal bodies) [34,35]. The presence of gems correlates with a decrease in coilin methylation [29,30]. Although the nuclear role of SMN is not well understood, it is possible that SMN escorts nascent snRNPs to the CB [6,8]. Interestingly, coilin can compete with Sm proteins for binding sites on SMN [28], indicating that locally high concentrations of coilin within the CB may serve to displace snRNPs from the SMN complex. The interplay between SMN, snRNPs and coilin may therefore regulate snRNP accumulation within the CB. We set out to determine if we could identify factors that control the departure of nascent snRNPs from the CB.

In this report, we demonstrate that an isoform of the human mitochondrial Tim50, Tim50a, localizes to speckles and interacts with snRNPs, SMN and coilin. Human Tim50, which has phosphatase activity, is a component of the mitochondrial translocator and regulates mitochondrial integrity and cell death [36]. The protein sequence of Tim50 and Tim50a are identical, with the important exception that Tim50a contains an alternative translational start sequence that adds 103 aa to its N-terminus relative to Tim50. Competition binding experiments show that SmB' does not compete with SMN for Tim50a binding sites, but SmB' does reduce coilin interaction with Tim50a. Furthermore, SMN and coilin compete for Tim50a binding sites and Tim50a forms a more efficient complex with snRNPs in vivo compared to Tim50. Based on these results, we propose that Tim50a is involved in the regulation of snRNP biogenesis and possibly the activity of nuclear SMN.

## Results and discussion

### Isolation of Tim50a

Given the central role of coilin in the formation and composition of CBs, especially in its ability to recruit the SMN complex and snRNPs, we wanted to identify proteins that interact with coilin and possibly regulate SMN and snRNP localization in the CB. Towards this end, we conducted a yeast two-hybrid screen with coilin as bait. A human brain cDNA library was chosen for this screen because we are interested in assessing CB protein dynamics in neuronal tissue, which is the cell type primarily affected in Spinal Muscular Atrophy. The C-terminal 214 aa of coilin, which interacts with SMN and Sm proteins, was the bait for the screen [28] (our unpublished observations). Consequently, other proteins that bind C214 may regulate the interplay between coilin/SMN and coilin/Sm and thus play a role in CB dynamics.

**Figure 1:**
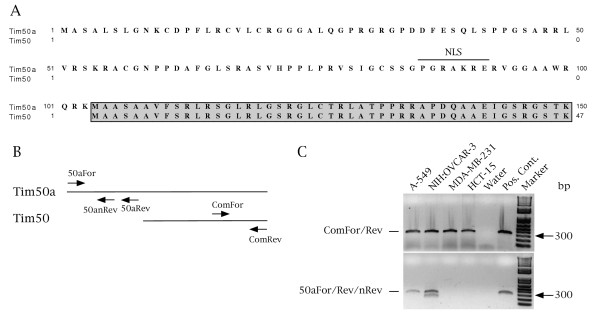
**N-terminal amino acid alignment and relative expression of Tim50a and Tim50**. (A) Compared to Tim50, Tim50a contains additional sequence that contains a putative nuclear localization sequence (NLS) upstream of the translational start of Tim50. (B) Schematic representation (not to scale) of Tim50a and Tim50 showing the locations of the primers used in the PCR reactions. (C) The relative expression of Tim50 versus Tim50a in four cancer cell lines. (Upper panel) A standard PCR reaction using cDNA from each cell line as template and ComFor+ComRev primers yields a product that could be from Tim50 and Tim50a. Water serves as a negative control while Tim50a plasmid cDNA serves as a positive control. Nested PCR (lower panel) demonstrates that Tim50a is detected in two cell lines but accounts for only a small fraction of the products from the *TIMM50 *gene. The cDNA from the following cell lines was used: A-549 (non-small cell lung cancer), NIH: OVCAR-3 (ovarian cancer), MDA-MB-231 (breast cancer) and HCT-15 (colon cancer). The lower band in the NIH: OVCAR-3 nested PCR reaction is a non-specific product.

Several preys were recovered from the screen, one of which was termed CAP50. Sequencing of this prey revealed that it contained the partial coding sequence for a protein known as Tim50. Since Tim50 localizes to the mitochondria and cytoplasm [36], the relevance of CAP50 interaction with coilin, a nuclear protein, was not initially clear. However, upon conducting EST (expressed sequence tag) database searches, we found that there are two possible translational starts for the *TIMM50 *gene product. The majority of ESTs terminate slightly upstream of the published translational start sequence of Tim50 (MAASA, [36]), suggesting that Tim50 is the major product of the *TIMM50 *gene. However, three ESTs, all from cancer cells lines, demonstrate that an alternative translational start sequence can be utilized which would add 103 aa to the N-terminus of the Tim50 protein. We have named this protein Tim50a (Figure 1A). Importantly, a putative nuclear localization signal (NLS) [37] is found within this region, indicating that Tim50a, unlike Tim50, may reside in the nucleus.

To verify the existence of Tim50a in other cell lines and to obtain a general idea as to the abundance of this transcript relative to the Tim50 isoform, we performed nested PCR with primers specific to Tim50a on cDNAs from four additional cancer cell lines: A-549, NIH: OVCAR-3, MDA-MB-231 and HCT-15. Another primer set was used in a standard (not nested) PCR reaction to amplify a region common to both Tim50a and Tim50 (Figure 1B). As shown in Figure 1C (upper panel), a standard PCR reaction (30 cycles) using primers that can bind both Tim50a and Tim50 (ComFor+ComRev) generate a product from all of the cell lines tested. In contrast, standard PCR reactions using Tim50a-specific primers (50aFor+50aRev) fail to yield a product from cell line cDNAs, but successfully generate a product using a positive control plasmid template (our unpublished observations). However, nested PCR (20 cycles) reveals that two of the four cell lines, A-549 and NIH: OVCAR-3, contain the Tim50a transcript (Figure 1C, lower panel), as monitored by sequencing of the product. The lower band in the NIH: OVCAR-3 reaction is from an unrelated transcript. Therefore, while Tim50a mRNA is not nearly as abundant as the Tim50 message, this species is present in at least four different cell lines (two lines from this study and two from the EST database).

### Cellular localization of Tim50a

**Figure 2:**
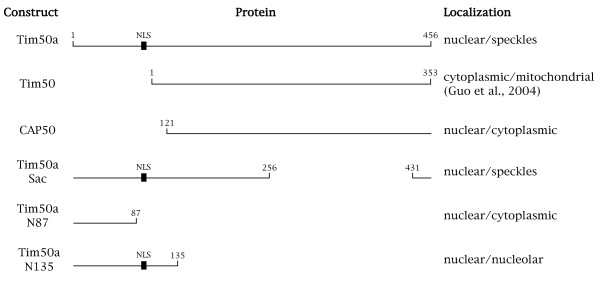
**Schematic representation and cellular localization of Tim50a constructs used in this study**. The Tim50a protein is 456 aa in length. Note that Tim50, reported by [36], lacks an NLS, which is present in Tim50a, Tim50aSac and Tim50aN135. Tim50aSac contains a deletion between aa 256 and 431.

**Figure 3:**
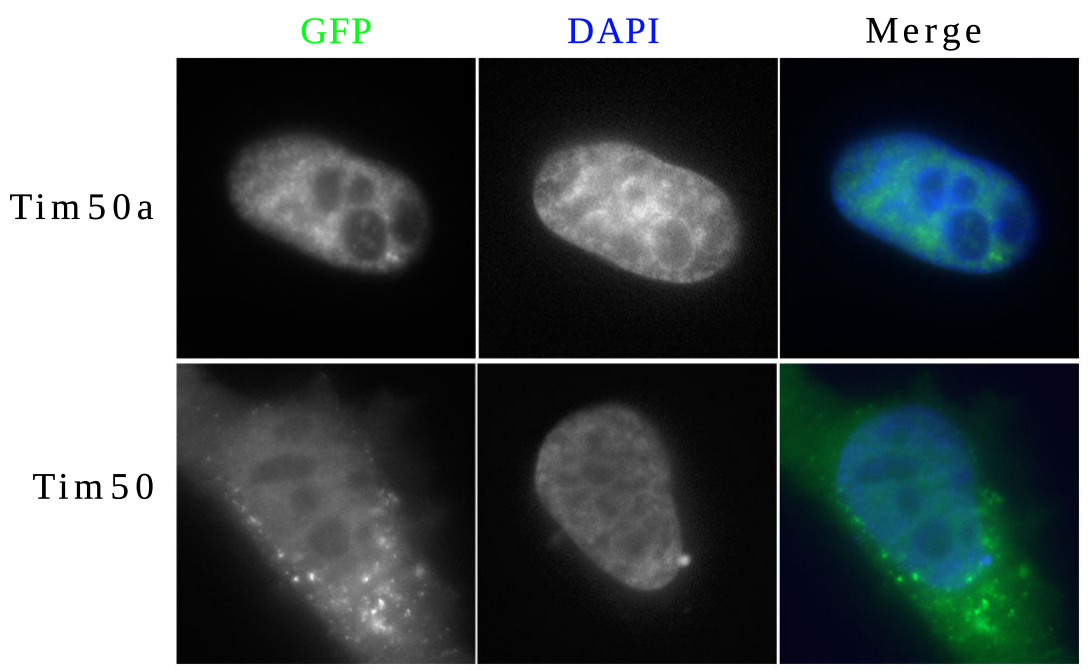
**Cellular localization of GFP-tagged Tim50a and Tim50**. HeLa cells were transfected with GFP-Tim50a (top row) or GFP-Tim50 (bottom row) and the cells were stained with DAPI to define the nucleus. The right panels show the overlay of the GFP signal (green) with the DAPI signal (blue). Note that GFP-Tim50 has extensive cytoplasmic staining but Tim50a is exclusively nuclear. The nuclear signal for GFP-Tim50 was not reported by [36] and may be caused by overexpression and/or fusion of GFP to the N-terminus of the protein.

**Figure 4:**
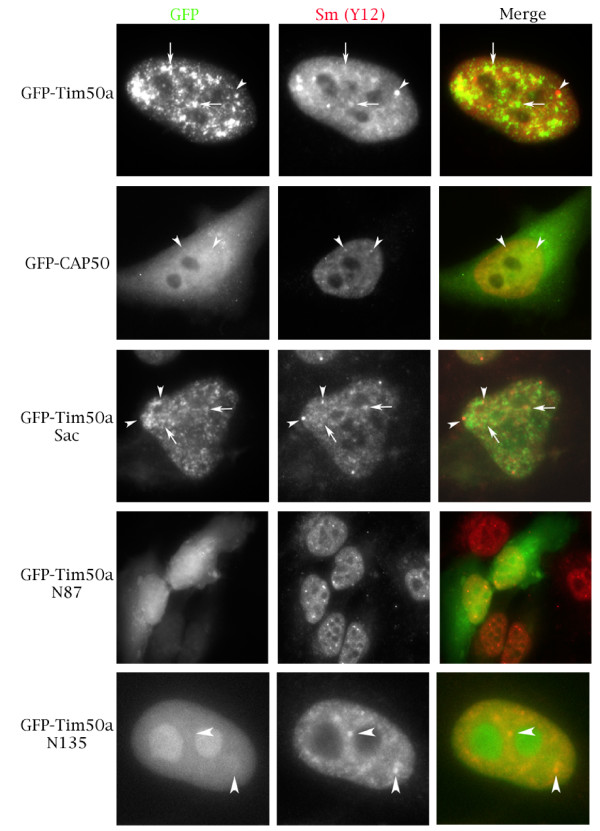
**Cellular localization of various Tim50a proteins**. Hela cells were transfected with GFP-tagged proteins (first column), followed by staining with antibody Y12 (second column) to detect Sm proteins (snRNPs). The merged image in color is shown in the last column (GFP is green, Sm proteins are red). Arrows mark speckles whereas arrowheads demarcate Cajal bodies. Note that cells for GFP-CAP50 and GFP-Tim50aN87 are shown at a lower magnification compared to the other images in order to demonstrate the cytoplasmic localization of these proteins.

To test if Tim50a is a nuclear protein, we obtained an EST encoding the additional residues and cloned the entire Tim50a coding sequence into a GFP expression vector. We also made a GFP fusion to the truncated Tim50 protein isolated from the two-hybrid screen (CAP50) as well as GFP fusions to various mutants of Tim50a. The localization of these proteins, and GFP fused to the mitochondrial Tim50 isoform, was monitored after transient transfection into HeLa cells (Figures 2 and 3). In contrast to Tim50, GFP-Tim50a is exclusively nuclear with speckle-like accumulations (Figure 3). To confirm that the nuclear accumulations are indeed speckles, in which snRNPs are stored, we stained the cells with antibodies against Sm proteins (Y12). There is overlap between Tim50a and snRNPs in speckles (arrows), as shown by the yellow-orange signal in the merged image (Figure 4, row 1, right panel). We do not detect Tim50a in Cajal bodies (arrowheads), indicating that the association of Tim50a with CBs or coilin is weak or transient. Alternatively, coilin may associate with Tim50a in the nucleoplasm. Indeed, while coilin is highly enriched in the CB, the majority (70%) of the protein is diffusely localized throughout the nucleoplasm [38]. We observed identical localization patterns for Tim50a upon fusion to a smaller myc-tag (our unpublished results), suggesting that the GFP-tag does not affect localization. The diffuse cytoplasmic and nuclear localization of GFP-CAP50 and GFP-Tim50a(N87) demonstrate that the region containing the putative NLS is necessary for nuclear localization (Figure 4). Tim50a mutants that retain the putative NLS display nuclear accumulation. For example, an internal deletion of Tim50a (Tim50aSac) is found in a speckled nuclear pattern while the first 135 aa (Tim50aN135) is nuclear and nucleolar. Tim50a localization to speckles is therefore contingent upon the NLS and residues found between aa 135 and 256.

### Actinomycin D redistribution of Tim50a

**Figure 5:**
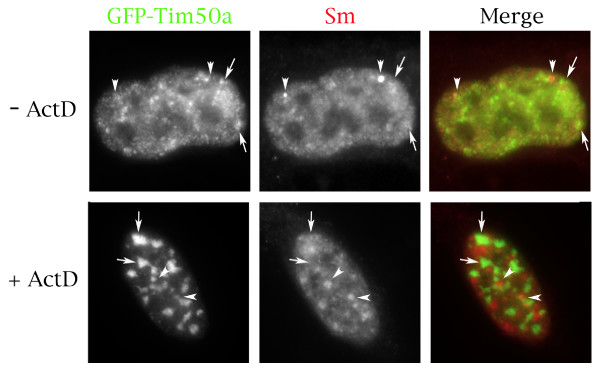
**Actinomycin D treatment alters the co-localization of Tim50a with snRNPs**. HeLa cells transfected with GFP-tagged Tim50a (green) were untreated or subject to actinomycin D, followed by fixation and staining with the anti-Sm antibody Y12 (red). The overlay of the images is shown in the last column. For untreated cells, arrowheads mark the location of Cajal bodies and arrows show speckles. For actinomycin D treated cells, arrows mark accumulations of Tim50a while arrowheads show enlarged Sm speckles.

The transcription inhibitor actinomycin D can be used to partially characterize the interaction of proteins in the nucleus. This is because actinomycin D inhibits transcription and causes the reorganization of splicing factors and Cajal bodies [39]. In particular, speckles enlarge and CBs are relocalized to the periphery of the nucleolus in the presence of actinomycin D [39]. If Tim50a were an integral part of speckles, then we would suspect that this protein would be found in enlarged speckles when cells are treated with actinomycin D. However, in the presence of actinomycin D, Tim50a no longer localizes with snRNPs in speckles but is instead found in discrete structures (Figure 5, bottom panels, arrows). This is evident in the merged image, which shows separate green (GFP-Tim50a, arrows) and red (snRNPs, arrowheads) signals with little overlap. In contrast, non-treated cells display overlap between Tim50a and snRNPs, resulting in a yellow-orange signal in the merged image (Figure 5, top panels). We tested whether Tim50a in actinomycin D treated cells localized to paraspeckles, another subnuclear domain of unknown function [40]. No colocalization of Tim50a with the paraspeckle marker protein, PSP1, was observed (data not shown). Taken together, the localization data indicate that Tim50a is likely to have a different function compared to Tim50, possibly in the regulation of CB activity or snRNP biogenesis. However Tim50a is not an integral component of speckles given the separate localization pattern of Tim50a and snRNPs in actinomycin D treated cells.

### Direct interaction of Tim50a with coilin, SmB' and SMN

**Figure 6:**
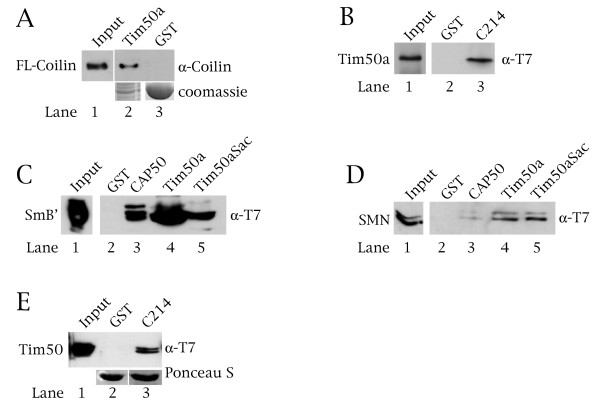
**Tim50a directly interacts with coilin, SMN and SmB'**. GST-pulldown assays were conducted on bacterially purified proteins. (A) Tim50a interacts with coilin. Soluble coilin was incubated with beads loaded with GST or GST fused to Tim50a. After incubation and washes, the reactions were subject to Western blotting and probing with antibodies to coilin. The input lane accounts for 26% of the coilin used in the pulldown reactions. The bottom panel is a coomassie stained gel demonstrating the levels of GST-Tim50a (lane 2) and GST (lane 3) used in the pulldown assay. (B) Tim50a interacts with the C-terminus of coilin. Soluble TV-tagged Tim50a was incubated with beads loaded with GST or GST fused to the C-terminal 214 aa of coilin (C214, the same portion of coilin used in the two-hybrid screen). After incubation and washes, the proteins were subject to Western blotting and probing with antibodies to T7. The input lane accounts for 20% of the Tim50a used in the pulldown reactions. (C) SmB' interacts with Tim50a and mutants thereof. T7-tagged SmB' was incubated with GST, GST-CAP50, GST-Tim50a or GST-Tim50aSac, followed by washing and probing with anti-T7 antibodies. The input lane accounts for 20% of the SmB' used in the pulldown reactions. (D) SMN interacts with GST-CAP50, GST-Tim50a and GST-Tim50aSac. Soluble T7-SMN was incubated with various GST fusions, followed by Western blotting and probing with anti-T7 antibodies. The input lane accounts for 10% of the SMN used in the reactions. (E) The mitochondrial Tim50 protein interacts with the C-terminus of coilin in vitro. Soluble T7-tagged Tim50 was incubated with GST or GST-C214 beads, followed by Western blotting and probing with antibodies to T7. The input lane (lane 1) accounts for 20% of the Tim50 used in the pulldown reactions. The membrane was stained with Ponceau S to verify that equivalent amounts of GST proteins were used in the assay.

The two-hybrid screen suggests that Tim50 isoforms may interact with coilin. To prove this, we conducted GST-pulldown assays using bacterially purified T7-tagged coilin and Tim50a fused to GST. Coilin is not recovered over beads fused to GST alone, but is recovered over GST-Tim50a beads (Figure 6A). We next tested if Tim50a would interact with the C-terminal 214 aa of coilin (C214), which is this same fragment of coilin used to isolate CAP50 in the two-hybrid screen. In agreement with the two-hybrid results, soluble Tim50a is recovered over GST-C214 beads, but not by GST alone (Figure 6B). Since Tim50a localizes to speckles, we were interested in assessing if Tim50a interacts with Sm proteins of snRNPs. Using SmB' as a representative of Sm proteins, we conducted GST-pulldown experiments with a variety of GST-Tim50a proteins. Consistent with the localization data, SmB' directly interacts with GST-Tim50a (Figure 6C, lane 4). SmB' also associates with two mutants of Tim50a, CAP50 and Tim50Sac. Based on the binding results, we suspect that the interaction domain for SmB' on Tim50a lies somewhere between residues 121 and 256 of Tim50a, although we cannot exclude the role of the residues at the extreme C-terminus of Tim50a (aa 431–456, Figure 2). Given that Tim50a localizes to speckles and directly interacts with coilin and SmB', we next tested if Tim50a could interact with another protein crucial for snRNP formation, SMN. As shown in Figure 6D, SMN binds to the GST-Tim50a proteins but not to GST alone. We also examined if the isoform known to localize in mitochondria, Tim50, also interacts with the coilin fragment C214 as found for Tim50a. Purified full-length Tim50 does indeed interact specifically with the coilin fragment as shown in Figure 6E. From these in vitro binding studies, we conclude that coilin, SmB' and SMN can interact with both Tim50a and Tim50 isoforms, but the interaction of these proteins in vivo is likely regulated by their subcellular localization.

### SmB' and SMN compete with coilin for Tim50a binding sites

**Figure 7:**
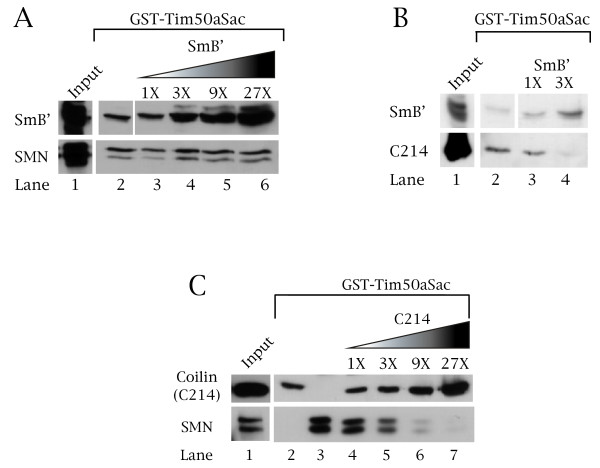
**SmB' and SMN compete with coilin for Tim50a binding sites**. Competition GST-pulldown experiments are shown. (A) SMN does not compete with SmB' for Tim50a binding sites. GST-Tim50aSac was incubated in separate reactions with SmB' (lane 2, top) or SMN (lane 2 bottom) followed by probing with anti-T7 (to detect SmB') and anti-SMN (to detect SMN). Lanes 3–6 show separate reactions in which a fixed amount of SMN was incubated with GST-Tim50aSac along with an increasing amount of SmB'. In lane 6, 27 times more SmB' was used in the reaction compared to the level of SMN. The input lanes account for 20% of the amounts used in all the reactions for SMN and the reactions shown in lane 2 and 3 for SmB'. (B) SmB' competes with coilin for Tim50a binding sites. GST-Tim50aSac was incubated with a fixed amount of the C-terminal coilin fragment (C214) and an increasing amount, in separate reactions, of SmB'. After Western blotting, anti-T7 was used to detect SmB' (top) and anti-coilin was used to detect C214 (bottom). Lane 4 shows the result of a reaction in which 3 times more SmB' compared to C214 was used in the binding assay with GST-Tim50aSac. Note that the level of recovered C214 is reduced in lane 4 compared to lane 2. The input lanes account for 20% of the amounts used in all the reactions for C214 and the reactions shown in lane 2 and 3 for SmB'. (C) Coilin competes with SMN for Tim50 binding sites. A competition experiment is shown wherein individual reactions were set-up containing a fixed amount of GST-Tim50aSac and SMN with increasing amounts of coilin fragment (C214). In lane 7, 27 times more coilin fragment was used in the reaction compared to the level of SMN. After SDS-PAGE and Western transfer, SMN and the coilin fragment were detected using antibodies specific to each protein. Lanes 2 and 3 show the binding of the coilin fragment or SMN to GST-Tim50aSac in the absence of competitor, respectively. The coilin fragment was added to the reactions containing GST-Tim5aSac and SMN starting at lane 4. As can been seen in lane 7, as more coilin fragment binds to GST-Tim50aSac, very little SMN is recovered. The input lane accounts for 20% of the amounts used in all the reactions for SMN and the reactions shown in lane 2 and 4 for the coilin fragment.

The ability of Tim50a to bind coilin, SmB' and SMN raises the interesting possibility that this protein may regulate the dynamics of CB proteins. To test this hypothesis, we conducted competition GST-pulldown experiments using GST-Tim50aSac. This protein was used instead of GST-Tim50a (Figure 2) because it is more easily purified compared to GST-Tim50a and has similar binding capabilities (Figure 6C and 6D). For the first experiment, we tested if SMN and SmB' vie for the same binding sites on Tim50a. This was accomplished by incubating a fixed amount of GST-Tim50aSac with a constant amount of SMN and, in separate reactions, an increasing amount of SmB'. Despite binding a large amount of SmB' (Figure 7A, top panel), the level of SMN recovered by the GST-Tim50aSac beads does not change (Figure 7A, bottom panel, compare the SMN level in lane 6 to that in lane 2). We conclude that Tim50a has separate binding sites for SMN and SmB' and that one protein does not preclude the other from binding.

We then tested if coilin and SmB' compete for binding sites on Tim50a. For this experiment, we incubated a fixed amount of GST-Tim50aSac with a constant amount of a coilin fragment (C214) and, in separate reactions, an increasing amount of SmB'. As shown in Figure 7B, increasing the level of SmB' decreases the recovery of the coilin fragment (C214) by GST-Tim50aSac (bottom panel, compare the amount of C214 recovered in lane 4 to that in lane 2). Thus coilin and SmB' compete for binding sites on Tim50a.

Finally we monitored if coilin and SMN compete for the same binding sites on Tim50a. This experiment was conducted with a fixed amount of GST-Tim50aSac and a constant amount of SMN with increasing levels of the coilin fragment (C214). The binding of the respective proteins to the Tim50a substrate in the absence of competitor (Figure 7C) is shown in lane 2 (upper panel) for the coilin fragment and lane 3 (lower panel) for SMN. In reactions with both soluble proteins, as the level of coilin fragment (C214) is increased, the amount of SMN recovered by the Tim50a beads is reduced (compare the SMN signal in lane 7 to that in lane 3). These data are consistent with a competition for Tim50a binding sites by SMN and coilin. Given its protein interaction potential, we hypothesize that Tim50a may affect CB function by interacting with proteins that localize to this domain. One possible scenario is that Tim50a displaces snRNPs from the CB and facilitates their translocation to speckles. Tim50a may also disrupt the interaction between coilin and SMN, allowing for SMN to leave the CB.

### Tim50a interacts with SMN and snRNPs in vivo

**Figure 8:**
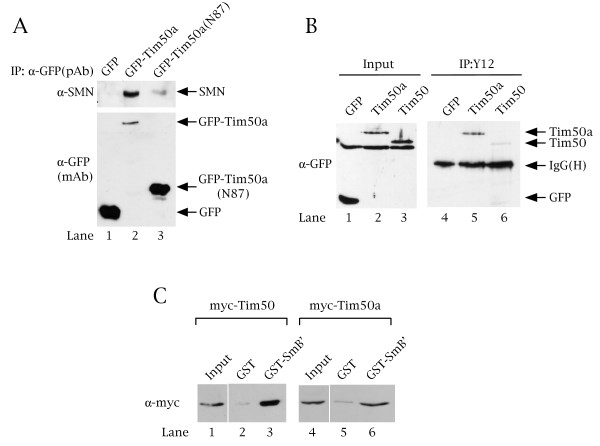
**Tim50a interacts with SMN and snRNPs in vivo**. (A) SMN and Tim50a form a complex in vivo. HeLa cells were transfected with empty GFP vector or GFP-tagged Tim50a and Tim50aN87. Lysate was subjected to immunoprecipitation with polyclonal anti-GFP antibodies, followed by Western blotting and probing with anti-SMN antibodies (top panel). The blot was reprobed with monoclonal anti-GFP (lower panel) to visualize the level of immunoprecipitated GFP, GFP-Tim50a and GFP-Tim50aN87. (B) Tim50a and Tim50 form a complex with snRNPs. HeLa cells were transfected with empty GFP vector, GFP-tagged Tim50a or GFP-Tim50 and lysate was subjected to immunoprecipitation with anti-Sm (Y12) antibodies, followed by Western blotting and probing with anti-GFP antibodies. Immunoglobulin heavy IgG(H) chains are marked. The input lanes represent 2% of the amount of lysate used in the immunoprecipitation reactions and have been overexposed five-fold relative to the immunoprecipitation reactions in order to visualize the GFP-Tim50a signal. (C) Tim50a and Tim50 bind the Sm protein, SmB'. HeLa cells were transfected with myc-tagged Tim50a or Tim50 and lysate was incubated with beads loaded with GST or GST-SmB', followed by Western blotting and probing with anti-myc antibodies. The input lanes represent 2% of the amount of lysate used in the pulldown reactions. Approximately equal amounts of GST and GST-SmB' proteins were used.

To corroborate the direct interaction data, we tested if Tim50a could associate with SMN in cells. For these experiments, we transfected HeLa cells with empty GFP vector, GFP-tagged full-length Tim50a or GFP fused to the N-terminal 87 aa of Tim50a. After incubation, lysates were made and subjected to immunoprecipitation with anti-GFP antibodies, followed by western blotting with anti-SMN antibodies. As shown in Figure 8A (upper panel), no SMN is co-immunoprecipitated with GFP alone but SMN is recovered by GFP-Tim50a. Only a faint SMN signal is observed in the GFP-Tim50a(N87) reaction (lane 3), suggesting that this fragment does not contribute significantly to the ability of SMN to bind Tim50a. Reprobing of this same blot with anti-GFP (bottom panel) demonstrates that while a large amount of GFP and GFP-Tim50a(N87) were immunoprecipitated, little SMN was recovered. In contrast, the amount of immunoprecipitated GFP-Tim50a is less than that obtained for GFP and GFP-Tim50a(N87), yet more SMN was recovered. Thus Tim50a can form a complex with SMN in cells.

Given that Tim50a localizes to speckles, in which snRNPs are stored, and directly interacts with a subunit of the Sm core, it is likely that Tim50a will interact with intact snRNPs. However, the in vitro binding data indicate that the mitochondrial isoform, Tim50, also has the potential to interact with snRNPs, which are known to have a cytoplasmic phase. To test this, we conducted a co-immunoprecipitation assay on HeLa cells transfected with empty GFP vector or GFP fused to Tim50a or Tim50. Lysates were generated and subject to incubation with the anti-Sm antibody Y12. After SDS-PAGE and Western transfer, the blot was probed with anti-GFP antibodies to monitor the association of GFP-Tim50a or GFP-Tim50 with snRNPs. As shown in Figure 8B, a signal corresponding to GFP-Tim50a is present in the Y12 immunoprecipitation reaction (lane 5). In contrast, less Tim50 is co-immunoprecipitated by Y12 (lane 6) compared to Tim50a. A GFP-alone signal is not observed in the Y12 reaction (lane 4), demonstrating the specificity of the reaction. Therefore, in vivo, Tim50a appears to be more efficient in snRNP interaction than Tim50, although we have not excluded the possibility that the GFP-tag may adversely affect Tim50 interactions. To address this point, Tim50a and Tim50 proteins were fused to smaller myc-tags, expressed in HeLa cells and tested for interaction with an immobilized Sm protein, SmB'. Both proteins bind GST-SmB', indicating that the GFP-tag may indeed contribute to the reduced interaction of Tim50 with snRNPs (Figure 8C). Alternatively, the faint Tim50 signal observed in the Y12 co-immunoprecipitation reaction (lane 6) may be an artifact due to the overexpression of GFP-Tim50 and subsequent nuclear localization, as shown in Figure 3. In support of this thought, immunofluorescence analysis of endogenous Tim50 demonstrates that this protein is predominantly cytoplasmic with little nuclear accumulation [36]. Given that Tim50a is a nuclear protein, we speculate that Tim50a interactions may regulate the nuclear activity of SMN and trafficking of snRNPs, possibly by altering the dynamics of the association between SMN, coilin and snRNPs. Additionally, it is plausible that cytoplasmic SMN can interact with Tim50 before mitochondrial import.

## Conclusion

The cellular localization and protein interaction characteristics of Tim50a suggest that this isoform of Tim50 may play a role in snRNP biogenesis. Based on the abundance of ESTs, it is likely that Tim50, not Tim50a, is the major product of the *TIMM50 *gene. However, the presence of Tim50a ESTs from cancer cell lines suggests that this protein may be induced in cellular transformation. It is known that cancer cells contain CBs, while some normal cells and tissues do not [41,35]. For example, normal adult lung tissue does not contain CBs, but lung cancer cells do. Since CBs play a role in snRNP biogenesis, it is likely that the increase of CBs in transformed cells reflects on the heightened demand in splicing requirements for upregulated genes that participate in the establishment and/or maintenance of the altered cellular physiology [42]. The induction of CBs may also facilitate the production of telomerase, the RNA component of which localizes to CBs [43,44]. In addition to cancer cells neuronal cells contain robust CBs [45], possibly because high levels of snRNPs are necessary to process the abundance of messages subject to alternative splicing in neuronal tissue [46]. Therefore, Tim50a expression may be limited to tissue with high demands in snRNP metabolism. We are currently developing an antibody to the unique N-terminus of Tim50a in order to explore this possibility. Additionally, experiments are underway to verify the role of Tim50a in snRNP biogenesis. In particular, we are interesting in determining if Tim50a is involved in the liberation of snRNPs from the CB after they have been modified. The functional consequence of the interaction between Tim50a and SMN is also being investigated. Finally we note that Tim50a contains the phosphatase domain shown to be functional in the Tim50 isoform [36], and thus may alter CB activity by modifying coilin, a known phosphoprotein [45].

## Methods

### Yeast two-hybrid screen, plasmid construction and mutagenesis

A human brain cDNA library cloned into the prey vector pACT2 and pretransformed into the yeast strain Y187 (BD Biosciences, Palo Alto, CA) was mated with the strain PJ69-2A harboring the bait vector pAS2-l-coilin(362–576, C214) per the manufacturer's instructions. After mating, the yeast were plated onto medium lacking tryptophan, leucine, histidine and adenine (to select for bait, prey and protein interaction). Colonies were picked after several days of incubation and the prey plasmid was isolated and transformed into PJ69-2A containing pAS2-1-C214 or control bait plasmids to confirm the specificity of the interaction. Restriction digests and sequencing revealed that nine different prey cDNAs were recovered, one of which was termed CAP50. CAP50 was sequenced and the results were used to conduct a BLAST search on the NCBI website. Based on this analysis, we concluded that CAP50 contained the partial coding sequence for the protein Tim50 [36]. Several expressed sequence tags (ESTs) corresponding to Tim50 are present in the database. One EST, BE386959, contains upstream sequence information not found in the majority of Tim50 ESTs or reflected in the Tim50 protein sequence. We procured EST BE386959 and used this cDNA as a template in a PCR reaction with primers designed to amplify the Tim50a isoform (*Bam*HI Forward primer: 5'GTCGGGATCCATGGCCTCAGCTTTATCTCT-3' and *Eco*Rl reverse primer: 5'-TCGGAATTCGAGGCCCAGAGTTCAGGGCT-3'). The TIM50a amplified product was cloned in frame into the pEGFP-Cl vector (BD Biosciences, Palo Alto, CA) at *Eco*RI + *Bgl*II restriction enzyme sites. Tim50a was also cloned in frame into pGEX-2T (Amersham Pharmacia, Uppsala, Sweden) and pET28a (Novagen, Madison, WI) vectors at *Eco*RI + *Bam*HI sites and utilized for bacterial protein expression. Various mutants of TIM50a were produced from specific restriction enzyme sites that were located within the TIM50a coding sequence or in pEGFP-C1. These constructs included TIM50aN87 (*Sma*I deletion), TIM50aSac (*Sac*I internal deletion) and TIM50aN135 (*Apa*I deletion). Also, CAP50 obtained from the yeast two-hybrid screen was digested with *Bam*HI + *Bgl*II. and cloned in frame into pGEX-2T at a *Bam*HI site and utilized for bacterial protein expression, or digested with *Bam*HI + *Eco*RI and cloned in frame into pEGFP-Cl at the equivalent sites for transient transfection experiments. The mitochondrial Tim50 was cloned into various expression vectors by standard techniques.

### PCR and nested PCR of Tim50 isoforms on cancer cell line cDNA

PCR reactions were performed in the DNA Engine PTC-200 Peltier Thermal Cycler (MJ Research, Watertown, MA). The cDNA from four cancer cell lines were kindly provided by Dr. Laree Hiser (The University of Mississippi, Medical Center): NIH: OVCAR-3 (ovarian cancer), HCT-15 (colon cancer), A-549 (non-small cell lung cancer) and MDA-MB-231 (breast cancer). These cDNA were shown by Dr. Hiser to be free of genomic DNA. Two sets of primers were used in the PCR reactions, the sequence of which can be provided upon request. The first set (ComFor+ComRev) was designed to amplify a region common to both Tim50a and Tim50, thus providing a method to monitor the total expression level of the *TIMM50 *gene. The second set of primers (50aFor+50aRev or 50anRev) bind specifically to Tim50a and not Tim50, thereby allowing the level of the Tim50a transcript to be assessed. A standard reaction, consisting of 30 cycles of amplification and the ComFor + ComRev primers, was used to amplify the common region of Tim50/Tim50a from cDNA. Plasmid containing the Tim50a cDNA (10 ng) and water served as positive and negative controls, respectively. To observe the Tim50a isoform, nested PCR had to be conducted. For these reactions, 30 cycles of amplification using the 50aFor+50aRev primers was conducted using the cDNA as template in a 25 μl reaction. Water and 10 ng of Tim50a plasmid served as negative and positive controls, respectively. 0.1 μl of each product, including the negative and positive controls, was then used as a template in a 25 μl reaction with a second pair of primers (50aFor+50anRev) for 20 cycles of amplification. The resultant products were cloned and sequenced to verify that they were derived from the Tim50a isoform. Nested PCR using NIH:OVCAR-3 cDNA consistently yielded two products. Sequencing demonstrated that the upper product was from Tim50a while the lower product was from an unrelated message with partial homology to the primers used in the nested PCR reaction.

### Cell Culture, Transfection, Immunofluorescence, and Immunoprecipitation

HeLa cells from the American Type Culture Collection (ATCC) were cultured, transfected with Superfect (Qiagen, Valencia, CA) and processed as described [28,29,47]. Where indicated, actinomycin D was used at 5 μg/ml for two hours. For immunofluorescence, cells were grown on chamberslides and fixed with 4% paraformaldehyde followed by the permeabilization with 0.5% Triton X-100. The permeable cells were then blocked with 10% normal goat serum for 30 min and probed with the corresponding primary and secondary antibodies. For immunoprecipitation, whole cell lysate was obtained from HeLa cells transfected with specific constructs. Cells were lysed in 50 mM Tris, pH 7.5, 150 mM NaCl, 1% NP-40,1% sodium deoxycholate, 0.1% SDS, 2 mM EDTA plus 1 tablet of complete protease inhibitor cocktail (Roche, Mannheim, Germany) per 50 ml lysis solution. The lysates were then repeatedly passed through a 25-gauge needle to shear DNA. The cell lysate was incubated at 4°C for one hour with primary antibody, followed by the addition of Sepharose beads (Amersham Pharmacia, Uppsala, Sweden) and an additional one hour of incubation. Both incubation periods were accompanied with gentle rotation. The beads were washed with 1 ml lysis solution 5 times, resuspended in 5X SDS loading buffer, boiled, and subject to SDS-PAGE and Western blotting as described [47]. Antibodies used for this experimentation include anti-SMN (BD Biosciences, Palo Alto, CA, catalog number 610646), anti-GFP (polyclonal, BD Biosciences, Palo Alto, CA, catalog number 632459; monoclonal, Roche, Indianapolis, IN, catalog number 1814460) and anti-Sm (Y12) (Lab Vision, Freemont, CA, catalog number MS-450-P).

### In vitro binding assays

GST-tagged and His-tagged constructs, after transformation into E. coil BL21(DE3)pLysS cells, were induced and purified as described [28]. In binding reactions, approximately 1 μg of His-T7-tagged protein was incubated with 1 μg of the GST-fusion protein in 1 ml of lysis buffer plus 2 mM DTT. After incubation for 1 hour at 4°C with gentle inversion, the beads were washed 5 times (1 ml each) with lysis buffer plus DTT, resuspended in 10 μl 5X SDS loading buffer, boiled and subjected to SDS-PAGE. Primary antibodies used included anti-T7 (Novagen, Madison, WI, 1:1000), anti-SMN (described above) and anti-coilin [48], 1:500). Competition experiments were performed as described [28], except that increasing amounts of SmB' or coilin fragment were used.

## Authors' contributions

HX conducted binding assays and made DNA constructs. ZBS did cell localization, immunoprecipitation studies and competition binding assays. MRII conducted some competition binding assays. MDH conceived the experiments, coordinated the study and wrote the paper.
